# What is the clinical evidence to support off-label rapamycin therapy in healthy adults?

**DOI:** 10.18632/aging.206300

**Published:** 2025-08-07

**Authors:** Jacob M. Hands, Michael S. Lustgarten, Leigh A. Frame, Bradley Rosen

**Affiliations:** 1 The George Washington University School of Medicine and Health Sciences, Washington DC, DC 20037, USA; 2 Metabolism and Basic Biology of Aging, Jean Mayer USDA Human Nutrition Research Center on Aging (HNRCA) at Tufts University, Boston, MA 02111, USA; 3 The George Washington University School of Medicine and Health Sciences, Washington DC, DC 20037, USA; 4 mTOR Medicine Practitioner, Los Angeles, CA 90212, USA

**Keywords:** rapamycin, aging, mTOR

## Abstract

Low dose rapamycin therapy has been proposed as a longevity candidate in healthy aging adults. We present a review of the evidence for low dose rapamycin and rapalog therapies in healthy human adults and model the findings of one cohort study using the PhenoAge model. Despite the preclinical evidence supporting the use of sirolimus to enhance mean and maximal lifespan, the data in humans have yet to establish that rapamycin, or its analogues, is a proven seno-therapeutic that can delay aging in healthy older adults. Rapamycin and rapalogs warrant further study with larger cohorts to better establish their contribution to human aging.

## INTRODUCTION

Rapamycin therapy is considered a promising approach for lifespan extension and the delay of age-related disease, with numerous preclinical studies documenting benefit [1–19]. These benefits have inspired some patients to seek rapamycin therapy from specialty practitioners. Yet, the clinical evidence of benefit associated with low-dose rapamycin use in healthy human adults has not been established, and there may exist signals indicating caution with off-label use at non-immunosuppressive doses.

Rapamycin was first isolated in 1975 from a bacterium, *Streptomyces hygroscopicus*, found in a soil sample on Easter Island. Researchers would later discover that it had anti-fungal, anti-tumorigenic, and immunomodulatory properties [20, 22]. In subsequent decades, researchers studying yeast resistance to rapamycin identified its biological target, named the mechanistic target of rapamycin (mTOR) [21, 22]. At present, the role of mTOR inhibition in age-related disease has been well-documented in multiple preclinical models [1–19]. The mechanistic target of rapamycin acts as an integral regulator of cell growth, autophagy, and division. Rapamycin’s ability to delay age-related disease has been replicated, most notably in the intervention testing program (ITP), where consistent extensions for median and maximum lifespan in both male and female mice have been identified [22].

While the benefit of rapamycin therapy has been demonstrated in non-human models, nonetheless, the clinical evidence for low-dose mTOR inhibitors as a therapy for extending lifespan or delaying the onset of age-related disease in healthy adults remains unestablished. Here, we provide a critical appraisal of studies evaluating low-dose rapamycin therapy in healthy adults and offer considerations for its potential use as an off-label longevity drug in humans.

### Clinical evidence in healthy participants

As the concept of aging as a modifiable risk factor for illness and death continues to gain traction, repurposing previously approved drugs, such as rapamycin, metformin, and acarbose to delay age-related disease, is becoming increasingly prevalent.

Longevity data in humans is difficult to acquire. Any well-designed trial that attempts to assess the longevity impact for any drug in people will be time consuming, expensive, and complicated by uncertainties in clinically valid endpoints. Since rapamycin is a generic medication, there is little incentive for any private group to fund such a study, which further complicates acquisition of high quality evidence with regard to low-dose rapamycin therapy. Accordingly, the clinical evidence evaluating low-dose rapamycin, or its analogues, in healthy participants is scant, with less than a dozen known trials exploring a variety of biomarkers, including immune function, protein synthesis, and hematologic parameters.

Evidence favoring low-dose mTOR inhibition was established by Mannick et al. (2014) using everolimus (RAD001), an mTOR complex 1 (mTORC1) rapalog, to evaluate markers of immune function in older adults. Mannick et al. evaluated a cohort of 218 healthy older adults receiving everolimus therapy, which was discontinued two weeks prior to influenza vaccination [23]. The results of this analysis suggested that low-dose everolimus therapy (0.5 mg/day and 5 mg weekly) induced a 20% increase in immune titers, while circulating T-cell inhibiting PD-1 positive CD4 and CD8 counts declined relative to placebo, a finding which is associated with enhanced T-cell function, or a more youthful immune phenotype [23, 43]. Rather than being immune suppressive, low-dose mTOR inhibition was associated with signals of enhanced immune function in this population. While safety parameters were acceptable, benign apthous ulcers were significantly more common in the treatment arm. The ability of mTOR inhibition (everolimus) in combination with an ATP-competitive kinase inhibitor with secondary mTORi effects (RTB101) in reducing respiratory tract infection was confirmed by Mannick’s group in 2018 in a phase 2 trial of 264 healthy adults [24]. Caution is appropriate in interpreting these results, as the study did not detect a significant difference in annualized rates of respiratory tract infection, though the study may have been underpowered to detect this [25].

In a follow-up phase 2b and phase 3 analysis, 10 mg/day of RTB101 demonstrated a similar effect and was associated with a marked reduction in respiratory tract infection incidence [25]. Laboratory analysis supported a phenotypic improvement in immune function, mediated by an upregulation of interferon-gamma (IFN-γ), an inflammatory cytokine whose induction stems from NF-kB activation. Several caveats apply in this trial. In the 2b phase, participants (n = 652) were initially randomized to RTB101 at 5mg/day, 10 mg/day, or placebo. Importantly, the incidence of respiratory tract infections did not differ significantly between the 5 mg/day dose versus placebo. In part 2 of this trial, investigators instead randomized the remaining participants (473) to 10 mg/day, 20 mg/day, 10mg/day + everolimus 10 mg/day, or placebo, noting that only the 10 mg/day dose of RTB101 was sufficient to reduce the respiratory tract infection rate relative to control. No significant safety events occurred and, similarly to the 2a trial, isolated mTORC1 inhibition was confirmed, and there were no significant differences in hyperglycemia or hyperlipidemia. The relative success of lower doses versus higher doses is of potential interest and may point towards the existence of an immunologic “threshold” at which the immune enhancing effects of low dose mTOR inhibition are replaced by immunosuppressive effects observed at higher doses.

Other limitations should be noted, including the increase in respiratory tract infection incidence in smokers or those with COPD. Although “intermittent” mTOR inhibition achieved a clinically significant effect, continuous therapy did not. In the phase 3 arm of this study, the results of the phase 2b study were not replicated, though this finding was complicated by an endpoint alteration from laboratory-confirmed infections in phase 2 to patient-reported infections in phase 3, as requested by the FDA. This decision muddied their findings, limiting the ability to detect a significant difference between groups. The authors suggested that this unexpected observation may be the result of improper symptom logging and/or a healthier cohort composition. The latter consideration may be significant because the effect of mTOR inhibition was greatest in subjects >85y, and in subjects >65y with evidence of asthma, when compared with cohort controls. Thus, rapamycin therapy may have an outsized, positive effect on hematopoietic parameters in immunologically-challenged participants. In sum, the evidence favoring the beneficial effect of rapamycin therapy on the incidence of respiratory tract infection is compelling, but not convincing at present.

Kaeberlein et al. (2023) reported on community use of rapamycin for longevity purposes, finding that users had a significantly lower likelihood of COVID infection and long-COVID incidence along with subjective increases in various measures of well-being and physical stamina [26]. This cohort of rapamycin users also documented self-reported benefit in abdominal cramps, depression, abdominal pain, muscle tightness, anxiety, and eye pain relative to non-users. It is important to note this cohort was not blinded to their intervention and may have been impacted by some degree of placebo effect. Importantly, this cohort reported no safety signals. Nonetheless, the Kaeberlein study supports observations made by previous human and animal studies with respect to immune enhancement. Thus, while promising, this study does not constitute firm evidence that rapamycin can extend healthspan or lifespan in humans.

Trials of rapamycin have yielded ambiguous evidence with respect to muscle protein synthesis. For example, Gunderman et al. (2014) demonstrated that 16 mg of rapamune blunted post-exercise increases in protein synthesis [27]. In contrast, Dickinson et al. (2013) found that the same dose did not alter rates of synthesis in skeletal muscle as assessed via muscle biopsy, nor did it affect circulating markers of autophagy up to 4 hours post-ingestion relative to baseline [28]. However, this study only considered basal post-absorptive and non-post-exercise rates. Thus, it is possible that sirolimus may demonstrate anti-sarcopenic effects that should be considered, particularly because sarcopenia has been repeatedly associated with adverse outcomes in the context of age-related decline [29, 30]. However, it is also plausible that muscle protein synthesis exerts age-dependent effects and that mTOR inhibition by sirolimus may in fact benefit other aspects of muscle preservation including reduced catabolic activity. The relative absence of surveillance and testing for this finding in other cohorts limits the conclusions derived from these two clinical studies.

In a trial by Horbelt et al. (2020), 22 healthy young men were subjected to RAD001 (everolimus) at various dosing schedules to evaluate its effect in 4 doses over 12-hour periods for 15 days [31]. Several days post-administration, significant reductions for two interleukins (IL), IL-2 and IL-10 were identified. Reductions in IL-10 are noteworthy and warrant attention given that IL-10 is recognized as a key anti-inflammatory cytokine, as it promotes homeostasis (resolution of inflammation) and immune surveillance (preparation for robust response given need, e.g. cancer cells or pathogens) [32]. IL-10 expression has also been associated with increased longevity [32]. With respect to psychological parameters, lower and medium doses (5-10 mg) of everolimus significantly increased self-reported anxiety and increased noradrenaline. While these changes cannot be interpreted in isolation, the net effect of rapamycin therapy in this cohort was significant and merits further consideration.

One of the most detailed trials of low-dose rapamycin use in healthy participants comes from Kraig et al.’s (2018) examination of 25 healthy older adults (aged 70-95 years), which evaluated the safety of 1 mg/day sirolimus for 8 weeks. A mean 7.2 ng/dL circulating level of sirolimus was achieved, with authors documenting changes in several hematologic, hormonal and physical parameters [33]. The limitations of this study are (1) a relatively short duration and (2) the use of continuous (1 mg/day) rather than intermittent rapamycin dosing schedules that may lead to off-target effects. Kraig et al.’s results reported no significant improvement in various metabolic parameters, while noting several potentially undesirable findings between groups, including a significant decrease in plasma albumin, increased triglycerides and Hemoglobin A1C (HbA1C) and a near-significant (p=0.06) increase for very-low-density lipoprotein (VLDL) within rapamycin-treated subjects. Albumin declines during aging [34], which could suggest an unfavorable age-related change with rapamycin use, though liver function biomarkers appeared unaffected. Increases in triglycerides, HbA1C, and VLDL are also concerning, however, insulin sensitivity was not altered, nor were results from oral glucose tolerance tests or fasting glucose. Thus, Kraig et al. concluded that rapamycin therapy did not lead to significant adverse outcomes in the short term. Alterations in various hematologic parameters on rapamycin therapy included a significantly decreased red cell distribution width (RDW), a marker that has been associated with a “youthful” biological age [35]. Other hematologic parameters were consistent with previous trials of rapamycin therapy that identified decreases for mean corpuscular volume (MCV), mean corpuscular hemoglobin (MCH), and hemoglobin (Hgb) [36–38]. A higher MCV and lower hemoglobin are associated with an increased all-cause mortality risk [36–38], which may suggest an opposing impact on health-related biomarkers. With regard to physical parameters, weight was reduced in the rapamycin cohort, however, handgrip strength was unchanged, which may support the complex role of mTOR inhibition in muscle preservation. There is also evidence that handgrip strength may be related to cognitive capacity, thus suggesting a possible protective role against neurological decline [39]. Further, though walking speed declined markedly in the control group, the rapamycin cohort maintained gait speed without a significant functional decline.

Rapamycin users demonstrated no significant reduction on any individual inflammatory marker in this small cohort. Instead, inflammatory cytokines broadly increased, including a significant TNF-α elevation. This cohort was not appropriately powered and thus non-significant changes in inflammatory parameters such as IL-2, IL-6, and monocyte chemoattractant protein 1 (MCP-1), while interesting, may not necessarily denote harm and instead could be artifacts of the study. As a caveat, TNF-α is a marker of autophagy; thus, we cannot conclude whether this finding is a confound associated with an underlying, healthy alteration [40]. Some circulating immune cells (CD) increased, with a signal indicating regulatory T cell (Treg) clusters may have improved if more appropriately powered. Treg expression is considered a marker of “healthy” tolerance, as it promotes homeostasis and immune surveillance [41, 42]. Further work to better understand the complex relationship between various immune components and aging is needed. Kraig et al. concluded that rapamycin therapy did not demonstrate any significant adverse outcome in the short term, nor was a signal of clear benefit identified.

### Theoretical evidence

Mendelian randomization provides a strong theoretical basis for potentially understanding the impact of biological therapy. In a preprint from Sobcyzk and Gat (2023), a genetically-predicted reduction in mTOR expression was correlated with an increased chance of attaining exceptional longevity. However, significant inverse associations were not identified for stroke, coronary artery disease, myocardial infarction (MI), or prostate cancer, but there was evidence of a reduction for heart failure risk [43]. Reduced mTOR signaling was also associated with a significant increase in genetically-predicted incident diabetes. Study authors also noted that mTOR was inversely associated with body weight, height, and predicted an earlier onset of female period and a lower basal metabolic rate.

Mendelian randomization studies have also evaluated the effects of downstream products of mTOR therapy, noting that its reduction may be associated with a lower incidence of Parkinson’s and Alzheimer’s diseases, for which there are currently limited treatments and a poor prognosis [44, 45]. These considerations are nuanced and may support a role for targeted rapalogs or their derivatives in the treatment of age-related disease.

### Modeling a sample cohort

The subject of clocks in human aging is controversial, with some authors documenting limitations in extrapolating the results of any “one” clock in terms of predictive capacity [46–48]. The following modeling analysis uses Levine et al.’s “PhenoAge” to explore the theoretical net effect of rapamycin treatment in the Kraig et al. trial. These data are suggestive at best and are limited by lack of subject level observations.

## Methods

To more precisely estimate the effect of the Kraig et al. sirolimus intervention, we modeled a series of patients representing a similar mean age (placebo, 80.6y; rapamycin-treated, 80.4y) pre- and post-intervention, and entered their average biomarker values into a Phenotypic Age calculator, a biomarker-based aging “clock” with a strong correlation (0.94) to chronological age, with an older PhenoAge associated with an increased all-cause mortality risk [46]. Both C-reactive protein (CRP) and the percentage of lymphocytes (LYMPH %) were not available in the Kraig et al*.* dataset; thus, we imputed age-expected values for both groups, which were held constant (CRP = 1.75 mg/L, LYMPH = 19%) [49–50]. Statistical significance for this comparison could not be determined owing to the lack of subject level data.

## Results and Discussion

In the placebo group, baseline and end-of-study Phenotypic Age were 78.32y and 78.47y, with a calculated Difference = +0.15y. In the rapamycin cohort, baseline and end-of-study Phenotypic Age were 81.34y and 77.38y, which is a net -3.96y change, which may be a significant finding when compared with the +0.15y PhenoAge change for placebo (Table 1). The rapamycin cohort, assuming no significant changes reported in LYMPH % or CRP, may have successfully reduced their estimated biological age with daily, low-dose rapamycin therapy.

**Table 1:** Example phenoage model.

Controls*	Rapamycin*
	
**Pre-trial**	**Pre**
Phenotypic age = 78.32y	Phenotypic Age = 81.34 y
Chronological Age = 80.6y	Chronological Age = 80.4y
Difference = -2.28y	Difference = + 0.94y
	
**Post**	**Post**
Phenotypic Age = 78.47y	Phenotypic Age = 77.38
Chronological Age = 80.4y	Chronological Age = 80.4
Difference = -1.93y	Difference = -3.96y
	
Control: Difference (Post) – Difference (Pre) = 0.15 years of Phenotypic Age	Rapamycin: Difference (Post) – Difference (Pre) = -3.96 years of Phenotypic Age

CRP and %LYMPH % were not available, chronological age-expected (CRP=1.75 mg/L, LYMPH = 19%) were imputed.

### Neurocognitive function

Results of neurocognitive trials using rapamycin have not been completed, however, several authors have provided evidence of plausible mechanistic benefit [51]. Currently there are two ongoing trials for rapamycin in the domain of cognitive function, such as a Phase 2 study on rapamycin for mild cognitive impairment (MCI) and early Alzheimer's (AD) and include brain imaging assessments (MRI and PET scans). These studies aim to evaluate potential structural changes, including gray matter metrics, over treatment periods.

“Evaluating Rapamycin Treatment in Alzheimer’s Disease using Positron Emission Tomography” (ERAP) or the ERAP trial is a six-month-long, single-arm, open-label, phase IIa biomarker-driven study which is testing rapamycin at a dose of 7 mg weekly and measuring changes in cerebral glucose metabolism [52]. The other trial is the “Rapamycin - Effects on Alzheimer's and Cognitive Health” (REACH) which aims to test the effect of 1 mg of daily rapamycin on various markers of AD disease burden [53]. Jointly, these trials may provide preliminary answers as to whether or not preclinical benefit can be translated into *in vivo* measurable benefit.

### Cardiovascular and cerebrovascular systems

The cardiovascular effects of rapamycin vary across vascular territories and cardiac function. While there is plentiful research on animal models and in tissue and cell models, human outcome data is sparse. While the use of biomarkers is no substitute for outcome data, under the circumstances the existing marker data is worth reviewing in this context.

Rapamycin has been associated with reductions in IL-10 and borderline increases in several pro-inflammatory biomarkers [31]. This is a finding that merits study. Further, while rapamycin elevates triglycerides and serum LDL at high doses in kidney transplant patients, CVD event risk was not increased [54, 55] and, in fact, stroke risk may have diminished. The local application of rapamycin as well as everolimus appear to reduce rates of restenosis and eventual CVD death after angioplasty compared to placebo [56, 57]. There is also limited evidence that rapamycin-treated patients are at lower risk for stroke [58]. In sum, there is insufficient human outcome data to comment on the impact of rapamycin therapy on human cardiovascular health.

### Cancer risk

There is no literature reporting cancer incidence or prevalence in healthy, non-immunocompromised cohorts with low dose rapamycin use, so inference from existing studies to an otherwise healthy cohort is challenging. In a meta-analysis of 20 randomized controls trials in kidney transplant patients, high-dose sirolimus treatment was associated with a lower overall kidney and NMSC cancer risk relative to other immunosuppressive regimens, but demonstrated no effect on other cancers with the exception of a potentially elevated prostate cancer risk. The authors of that study note the NMSC finding that this “may be partly due to removal of cyclosporine” as opposed to the addition of sirolimus [55]. Similarly, with reference to the elevation in prostate cancer risk the causality of this association is unclear and authors report that the association may be an artifactual and potentially related to rapamycin’s interference with PSA screening [55]. It is worth noting that findings of many preclinical models and approved clinical applications of rapamycin and or rapalogs have demonstrated potential mechanistic benefit with respect to cancer risk and treatment [59]. However based on outcome data in humans, we cannot comment on rapamycin’s cancer risk in healthy adults.

### Clinical implications and future directions

Despite extensive preclinical evidence supporting sirolimus and other mTOR inhibitors as potential gero-therapeutics, human data have yet to demonstrate that rapamycin can extend mean or maximal lifespan or delay the onset of age-related diseases. The findings reported herein underscore the need for larger, well-designed human studies to clarify rapamycin’s clinical relevance. Off-label prescribing should include vigilant monitoring for adverse effects and open discussion of knowns and unknowns with patients. Key priorities for future research include (1) establish efficacy with well-designed trials identifying relevant clinical endpoints in healthy adults, (2) identify therapeutic dose-response curves, and (3) explore synergistic interactions with other gerotherapeutics.

While once weekly dosing of 5-7 mg, or 10-15 mg biweekly is commonly recommended to patients, there is still no established dose response curve for rapamycin with regard to health span extension [60]. There are theoretical concerns with respect to intermittent dosing, including rebound mTOR hyperfunction following protracted mTOR inhibition. This concern may support a consistent weekly or daily dosing schedule as described in the only clinical trial that has been published with low-dose rapamycin in healthy older adults, but no longer term data exists to clarify this [33]. Rapamycin is estimated to impart biological effects at about 5 ng/mL and achieve greater relative toxicity above 15 ng/mL (pk parameters: 60-80 hours to 0.5 max concentrations), which suggests that rapamycin therapy (0.5-1 mg) daily or (5-7 mg) weekly could be sufficient to avoid side effects and reduce risk of theoretical hyper-mTOR functional reconstitution [61, 62]. mTOR inhibition offers an exciting and potentially important contribution to the biology of aging. However, rapamycin therapy in healthy participants remains incompletely understood, with limited outcome data.

## Conclusion

This paper has reviewed trials of low-dose mTOR inhibition therapy in human subjects. What emerges is a complex picture that remains insufficient to affirm or negate the longevity and healthspan extending benefits attributed to rapamycin. Despite the preclinical evidence supporting the use of sirolimus to enhance mean and maximal lifespan, the data in humans has yet to establish that rapamycin, or its analogues, is an effective seno-therapeutic to delay aging in healthy older adults.
